# Formative research to promote lupus awareness and early screening at Historically Black College and University (HBCU) communities in South Carolina

**DOI:** 10.1186/s41927-022-00323-6

**Published:** 2022-12-31

**Authors:** Edith M. Williams, Joni Nelson, Diane Francis, Keesha Corbin, Gary Link, Tomika Caldwell, Gary Gilkeson

**Affiliations:** 1 Department of Public Health Sciences, University of Rochester Medical Center, 265 Crittenden Blvd, CU 420708, Rochester, NY 14642 USA; 2 James B. Edwards College of Dental Medicine, Medical University of South Carolina, 73 Ashley Ave, BSB 127, Charleston, SC 29425 USA; 3 Department of Communication, University of Kentucky, 343 S. Martin Luther King Blvd, Lexington, KY 40506 USA; 4 Division of Rheumatology and Immunology, Medical University of South Carolina, 96 Jonathan Lucas Street, Charleston, SC 29425 USA

**Keywords:** Lupus, Awareness, African American, Qualitative research

## Abstract

**Background:**

Systemic lupus erythematosus or lupus is a severe chronic autoimmune disorder that disproportionately impacts young African Americans. Increasing lupus awareness in this high-risk group may be an effective approach to ultimately improving lupus outcomes. To begin to address this disparity, this report describes qualitative data to be utilized in the development of a campaign to enhance awareness of lupus on Historically Black Colleges and University (HBCU) campuses.

**Methods:**

Two focus groups (N = 14) were held with African American students in the network of HBCU’s in South Carolina to examine perspectives of focus group participants on knowledge, awareness, and experiences with lupus.

**Results:**

Five key emergent themes included: (1) Lupus Knowledge and Awareness, (2) Barriers for Not Seeking Healthcare, (3) Fatalism for Disease Burden, (4) Lifestyle Debilitation, and (5) Elevation of Education and Advocacy for Lupus. Additionally, five key recommendations emerged to improve lupus awareness and support, including: (1) remaining positive, (2) developing a supportive network, (3) the importance of increasing advocacy efficacy, and (4) messaging strategies around lupus, and (5) providing education to foster knowledge around the clinical impacts of lupus.

**Conclusion:**

Participants in our study stressed the necessity of lupus education and awareness among African American youth and expressed the desire for resources that would enable them to advocate for themselves and their families. Given the early age of onset for lupus, it is therefore vital to include African American youth in increasing education and awareness about lupus.

**Supplementary Information:**

The online version contains supplementary material available at 10.1186/s41927-022-00323-6.

## Background

*S*ystemic lupus erythematosus (SLE) is a severe disease with significant morbidity and mortality that impacts the African American community with greater frequency and severity than the White population, where diagnosis is often delayed or never made and mortality is significantly increased [1–5]. Racial and ethnic differences in prevalence (3–4× increased prevalence of lupus in African Americans), incidence, clinical features, disease activity, morbidity, mortality and disability from SLE are apparent [2, 4, 6–14]. Recent studies also demonstrated higher genetic risk [12], higher disease activity scores [10, 15], slower decline in disease activity [15], and a shorter time to renal involvement in Black SLE patients than White SLE patients [16, 17]. In addition, trends in SLE mortality indicate there is less progress in decreasing morbidity and mortality in African Americans than Whites [5, 18]. In addition, the disease has substantial impact on quality of life and productivity due to the early age of onset [19–24]. Lupus is a significant chronic disease in African American women. It affects primarily young women in their childbearing years (between the ages of 15 and 40 years). Progression to renal failure in young Black women with lupus is increasing and remains 5–10× that of White women [25, 26].

Multiple factors impact the evident health disparity in outcomes for African Americans with lupus. Though there are clear genetic reasons for the increased prevalence of lupus in African Americans compared to Whites [2, 27] as well as for progression of renal disease (i.e., apolipoprotein A1), socioeconomic factors are tightly entwined with race, making it difficult to determine causation of the increased morbidity and mortality in African Americans with lupus. Differential access to health care and therapies almost certainly plays a role [5, 23, 24, 28–30]. We cannot change the genetics of disease, but we can perhaps address in a meaningful way, awareness, access to care and adherence to physician appointments and medication regimens. Educating the community and their physicians regarding the diagnosis is a step towards improving outcomes in SLE [31, 32]. Most prior efforts [33], including our own, to enhance lupus awareness were through churches and community forums. The significance of lupus to the African American community is highlighted by recent initiatives by the Office of Minority Health to increase awareness of lupus through an educational program to enhance practitioner awareness of lupus and the recent IMPACT (Improving Minority Participation in Clinical Trials) grant awarded to the Lupus Foundation of America to develop methodology and tools to enhance minority participation in clinical research. Although these approaches do reach the community, they have not directly approached those at highest risk of lupus, young African American women. The current study used the network of Historically Black Colleges and Universities (HBCUs) in South Carolina to reach a large relatively untapped group as an approach to improving ethnic disparities in lupus. Two focus groups were held with African American students to obtain data on thematic issues relevant and specific to the types of messages that the target population would be most responsive to. This data is expected to inform development of a campaign to enhance awareness of lupus through outreach programs on HBCU campuses that will emphasize early signs and symptoms of lupus and provide resources for obtaining diagnosis and treatment.

## Methods

### Study design

Health belief model. Rosenstock, I. M. (1974). Historical origins of the health belief model. Health education monographs, 2(4), 328–335. Based on the health belief model (HBM), a public health framework, there are varying levels of influence that can impact individual beliefs about disease and illness. The HBM constructs include: perceived susceptibility of the disease, knowledge, awareness and other beliefs and lived experiences that can shape health behavior action (see Additional file 1: Fig. S1) [34]. Using constructs from the HBM, we conducted two focus groups (*n* = 14) to explore perspectives of young African Americans (AAs) (ages 18–24) about lupus. Study materials (i.e. interview guide and recruitment materials) were developed and Institutional Review Board (IRB) of the Medical University of South Carolina granted exemption in September 2019.

### Study participants

Six South Carolina HBCUs were invited to participate in the study, including: Allen University, Benedict College, Claflin University, Morris College, South Carolina State University, and Voorhees College. Historically Black Colleges and Universities (HBCUs) have an African American student population averaging 80%, and enroll about 25% of all African Americans seeking postsecondary education. Allen University and Benedict College are located in Columbia, South Carolina, directly adjacent to one another. Allen University is a private, coeducational HBCU connected to the African Methodist Episcopal Church with a very strong presence in the African American community. Benedict College is the fourth largest private HBCU in the US. South Carolina State University and Claflin University are located in Orangeburg, South Carolina, approximately 40 miles from Columbia. South Carolina State University is South Carolina’s first public four-year college for Black youth, and has played a key role in the education of African Americans in the state and nation. Claflin University is the oldest HBCU in South Carolina. Voorhees College is located in Denmark, South Carolina. Morris College is located in Sumter, South Carolina, approximately 45 miles east of Columbia. It has been a center for training ministers and teachers of the state and the nation, and is one of the few senior colleges built and operated solely under African American sponsorship. Table 1 shows projected student populations at the time of the study for the six campuses, totaling 10,400 persons aged 18–25. Students from each institution were invited to participate via recruitment flyers and digital messages posted in student health centers and other common areas of HBCU campuses. Recruitment information was also disseminated through each HBCU campus website, email, cell phone and existing text messaging systems. Both iterative and criterion sampling approaches were employed for the recruitment of participants. Students who indicated a willingness to participate and met the inclusion criteria were invited to participate. Participant criteria included: (1) African American race/ethnicity; (2) between 18 and 24 years of age; and (3) family history of autoimmune disease or current clinical diagnosis of SLE from a physician, according to ACR revised criteria for SLE [4]. While recruitment efforts were focused on HBCU campuses, participants did not have to be a current HBCU student to participate.

**Table 1:** Year 2020 student projections for SC HBCU campuses

Institution	Total population	Freshman population	Average age of freshmen	Male/female ratio
SC State University	4297	1112	18 years	40/60
Claflin University	2100	530	18 years	80/20
Allen University	698	227	22 years	46/54
Benedict College	2165	800	18 years	49/51
Voorhees College	491	149	18–24 years	43/57
Morris College	649	196	18 years	36/64
Total: 6	10,400	3014	N/A	N/A

This approach ensures maximum variation and reaching a point where no new themes emerged [35, 36]. Nineteen young African American expressed interest in participating during the recruitment period, and 18 young African Americans, representing two HBCU’s (Voorhees College and Claflin University) agreed to participate.

### Data collection

Focus groups were conducted to examine perspectives of focus group participants on knowledge, awareness, and experiences with lupus. Focus groups were conducted face-to-face or videoconference by experienced facilitators and were audio-recorded.

The first focus group was conducted in March, 2020. Of the 8 young African Americans who agreed to participate, 4 were in attendance; 3 females with lupus and one male with a family member with lupus. Three of the attendees attended an HBCU (one male and two females). The focus group was held in person in a conference room in the Department of Public Health Sciences at MUSC and via webex. One participant attended in person and three participants attended via webex. An experienced facilitator/navigator moderated the focus group discussion under the guidance of the supplement Co-PI. The meeting lasted two hours and in-person attendees were provided with lunch and a $25 cash incentive. Virtual attendees were mailed a $25 clincard. The second focus group was held in June, 2020. There were ten participants, ranging from 18 to 23 years of age, in attendance; 4 females and 6 males. Half of the participants attended Voorhees College, and the focus group was held in person in a classroom on the campus of Voorhees College. Two experienced facilitators moderated the focus group discussion under the guidance of the supplement Co-PI. The meeting lasted one hour and attendees were provided with snacks and a $25 cash incentive. Both focus group discussions were recorded and thematic analyses undertaken. Each focus group utilized a semi-structured interview guide that was developed specifically for this study and not published elsewhere. Areas of interests for the semi-structured interview guide were documenting participant experiences with lupus, influences on knowledge and attitudes about lupus, and effective messaging for lupus information (see Table 2, which shows a sample of the interview guide, and supplementary file of the entire interview guide).

**Table 2:** Focus group question and aim

Participant recommendations to improve lupus awareness and social support	Recommendation description
1. Different people may know or have heard different things about lupus, so I am interested in what you know. When you hear the word “lupus”, what comes to mind?	Gauges lupus awareness
2. What are the most important things you think people should know about lupus?	Gauges lupus awareness
3. Who do you think is most affected by lupus?	Gauges lupus awareness
4. What experiences or information have influenced what you think or know about lupus?	Captures the type of information and/or mode of delivery that was memorable and shaped opinions about lupus-based on personal experience(s)
5. What types of activities do you believe really work to increase awareness and understanding about lupus?	Captures the type of information and/or mode(s) of delivery that are memorable and opinion-shaping in general-could be based on personal experience(s) or other evidence, factual or perceived
6. What specific messages and/or ways of sharing information do you believe resonate with young African Americans?	Captures the type of information and/or mode(s) of delivery that are particularly memorable and opinion-shaping for young African Americans-could be based on personal experience(s) or other evidence, factual or perceived
7. What types of things could keep people from being aware of or understanding lupus?	Captures barriers to information consumption-could be based on personal experience(s) or other evidence, factual or perceived
8. If you or a family member has lupus, what are some of the most difficult aspects of your or their disease experience?	Captures critical disease components to include in an educational campaign-based on personal experience(s)
9. Is there anything that we haven’t talked about, but you feel is important to consider in educating young African Americans about lupus?	Captures any perspectives that may have been missed
10. Thank you for your participation. Do you have any questions, comments, or concerns?	Captures any perspectives that may have been missed

### Data analysis

Focus groups were analyzed using the constant comparative method [35–37]. This process includes identifying and categorizing concepts found in the text (open coding), aligning categories with one another (axial coding), and defining the final category, which supports the clarification of the phenomena (selective coding). Following this, operational definitions are developed for each coding category using relevant theoretical principles from HBM. We visualized matrices, using MAXQDAplus^®^, a qualitative data software, to recognize similarities and differences between codes, recurrence of codes, identify categorical patterns, and organize emergent themes [38]. The reliability and validity of the qualitative analysis were enhanced through iterative data collection, participant member checks and ongoing peer debriefing meetings with three experts in qualitative methods for examination and feedback. This research design permits a comprehensive description of the current narrative and elements that will contribute to the development of lupus education and awareness campaigns. The reliability and validity of the analysis was enhanced through iterative data collection, ongoing peer debriefing meetings with project director and three experts in qualitative methods for examination and feedback [35, 36].

## Results

Thematic analysis revealed focus group participants’ health beliefs around lupus and emergent themes were directly aligned with constructs of the HBM. Five key emergent themes included: (1) Lupus Knowledge and Awareness, (2) Barriers for Not Seeking Healthcare (3) Fatalism for Disease Burden, (4) Lifestyle Debilitation, and (5) Elevation of Education and Advocacy for Lupus.

### Theme 1: Lupus knowledge and awareness

All participants described their overall knowledge and awareness of lupus. Specifically, participants emphasized sources of identifying information about lupus and their perceptions about the risks for developing lupus. Additionally, participants were all in agreement that SLE is a systemic, non-curable, autoimmune disease that can impact multiple tissues in the body and cause varying symptoms per individual case.

The source of a majority of lupus knowledge was from a combination of family (*n* = 8), peer and community interaction (*n* = 6), and the internet (*n* = 2). Those that did not have a family member and/or peer who was previously diagnosed with lupus generally had little to no knowledge of lupus (*n* = 4). A few participants stated,

*“Before I was diagnosed, I had never heard of lupus.”*—Participant #7

*“My mom has it so I learned a lot about it from her…”*—Participant #4

“*I have a specialist that I see and if not, I look it up online. The lupus foundation website and other people in general who have lupus, we talk about it all the time*”—Participant #2

Participants also had limited knowledge on specific reasons for the cause of lupus and the other clinical conditions as a result of lupus (*n* = 4). Common perceptions about lupus causes included: genetics and the environment (e.g. staying in the sun too long), a few participants stated:

*“I don’t know how because no one in family got it, I am not sure if it was sun or not.”*—Participant #2

*“I was told that it could be inherited, by your environment, that’s what I was told.”*—Participant #1

### Theme 2: Barriers for not seeking healthcare

Some participants shared perceptions around barriers to seeking care for lupus-related symptoms (*n* = 3). Overall participants described barriers as stigma in the AA community in regard to visiting and trust in the healthcare system, the use of alternative home remedies for lupus symptoms, lack of transportation, inaccurate myths and limited knowledge about lupus. A few participants stated,

*“I know sometimes there is a stigma in the AA community and they are afraid to go to the doctor, or say something because they are afraid of the results…Or even if it’s not lupus, maybe a rash or something, just bringing awareness can help them to go get checked out and not wait too late. It’s a timing thing, you have to take care of your body and talk about other health things and tie lupus into it.”*—Participant #2

*“I have also heard about pregnancy and can’t conceive because the body becomes so inflamed. I have heard lots of wild things, like it causes some types of cancer. I think they are myths [because other I know have been] diagnosed and they have had children.”*—Participant #3

*“I know growing up for me, with my mom, sometimes people aren’t really educated and they just think things can go away with aspirin or creams, at home remedies, so we don’t create a bill and doctors tell you the same thing I am telling you or hope for the best.”*—Participant #2

*“Being educated and amenities available to people, like nearby hospital or healthcare professional and could add to wanting to get checked out.”*—Participant #4

### Theme 3: Fatalism for disease burden

Perspectives for overall health outcomes for AAs were described as ‘poor’ and not surprising for AAs to be at higher risk for lupus *(n* = 4). Participant revealed,

*“Everything affects [AAs] us”*.—Participant #2

*“We are God’s chosen people and we are given things to eventually affect us. People do all sorts of things to stress the body which make the immune systems weak and more likely to catch a disease.”*—Participant #6

In contrast, some participants stated that awareness and education should remain a priority for more than just AA communities.

*“Lupus can affect anybody and is not limited to just AAs”*—Participant #2

*“It would be better for awareness, and not to scare anyone but to be in the know about it, regardless of color and culture. Some people may think I can’t catch it because I am not of that race, but anyone can catch it.”*—Participant #2

### Theme 4: Lifestyle debilitation

Participant discussions also revealed their perspectives for the severity of lupus, including the social, clinical, and behavioral impacts of the disease. Participants described personal experiences or of other family members regarding multiple symptoms and disruptive impact on their daily living (*n* = 8)

*“Sometimes I get moody and I did lose all my hair”*—Participant #8

*“Joint pain in the body and headaches”*—Participant #6

*“Dry eyes and fatigue”*—Participant #5

*“When my body breaks out its frustrating, and don’t really like to go outside so not going out is not really a problem for me. I’m really tired a lot and break out a lot. I take 2 or 3 naps a day. Sometimes if I get sick, it takes a really long time for me to get back well.”*—Participant #2

*“One of biggest challenges was types of foods to change in the house with my family member (mom) with eliminating certain foods and seasonings to show support for her and increase my family’s physical health as well; more water less sugary juices and sodas.”*—Participant #4

*“Financially, she (mom) was the biggest breadwinner in the house so others had to take on other financial responsibilities to step up; it was not much of a problem because we were prepared for it but she felt like she was not doing enough for us and suffered from depression and so that was challenging.”*—Participant #3

### Theme 5: Elevation of education and advocacy for lupus

All participants described the necessity of education and awareness for AA youth about lupus. Varying formats of how, where and what lupus awareness information should be disseminated was discussed, some participants stated,

*“I would have a march to bring awareness”*—Participant #10

“*I would organize a school event*”—Participant #5 and Participant #2

*“I will be more of an advocate for my mother, I would tell when what I went through and make others more aware about it”*—Participant #6

*“Having an online mentor”*—Participant #3

*“I would agree, even a support group. My mom is a part of a support group and help to motivate her and have same lifestyles. A few people came out to talk about it at our church and we have an awareness month at our church. 15*–*20 minutes to talk about it, what it is, treatments available.”*—Participant #4

*“A lot of people around my age don’t really have an idea of what it is. We here about cancer and other ailments. When in school they had a lot of talks about STDs, like panelists, specialists, people that battle with it. It was an open table and no judgement”*—Participant #4

*“Media is one way and commercials with a lot of teenagers in them geared towards lupus and any other chronic disease that affects young adults too. That would be something that could be beneficial to get message out. Message has to be pertinent to me, relevant to me; more people are interested in the style of how its presented. Videos and seminars are perceived differently.”*—Participant #8

*“Reaching out to HBCUs, partnering with different organizations and you see a different audience depending on who is throws certain events. For example, I am in a sorority and people will attend based on what they think we will say. I think partnering with fraternities and sororities or different organizations on campus”*—Participant #2

Five key recommendations emerged to improve lupus awareness and support, including: (1) remaining positive, (2) developing a supportive network, (3) the importance of increasing advocacy efficacy and (4) messaging strategies around lupus and (5) providing education to foster knowledge around the clinical impacts of lupus. (Table 3. HBCU Participant Recommendations to Improve Lupus Awareness and Social Support). Overall, participants emphasized the significance of remaining positive about their lupus status to reduce negative impacts of physical symptoms, social support as a source of motivation and community, and reducing stigma of illness through education and advocacy through relevant messaging for youth.

**Table 3:** HBCU participant recommendations to improve lupus awareness and social support

Participant recommendations to improve lupus awareness and social support	Recommendation description
1. Positivity as the ‘new norm’	Promoting a positive environment for individuals living with lupus through empathy, encouragement, recognition of their needs and maintaining a hopeful outlook
2. Developing a supportive network	Forming a community network of family, friends, caretakers and allies (e.g. doctors, peer mentors, lupus patients, etc.) that serve as a support group for individuals living with lupus
3. Increasing advocacy efficacy	Equipping youth advocates with appropriate tools to reduce stigma surrounding lupus, receiving preventive screenings and clinical treatment, defining how to share appropriate messaging and champions for message delivery
4. Expanding messaging strategies	Increase the number of local lupus events, campaigns and social media messages and expand synonymous messaging between lupus and other chronic disease awareness efforts
5. Understanding clinical impact	Increase education on lupus symptoms, risk factors, decrease negativity surrounding physical limitations

## Discussion

We found that participants wanted health education and communication messages that delivered pertinent information about lupus. Such messages could be utilized to increase knowledge about lupus, address lupus-related stigma and fatalistic beliefs, and counter misinformation about what remedies work for people with lupus. Other important message elements could highlight the importance of knowing about lupus before someone may “need” such information. Many participants only encountered lupus information after they or a family member had been diagnosed. Participants also called for representation of African Americans alongside diverse audience members to reflect those at risk for lupus. Visuals with diverse audience members would address beliefs that lupus affects everyone and not just African Americans. Additionally, participants had suggestions for messages design approaches that should not be used during any campaigns. Specifically, they suggested not using scare tactics or fear-based appeals in the messaging. Participants found that focusing on messages that correct myths was a convincing approach to increase effectiveness of educational materials. Above all, lupus awareness messages targeted at African Americans need to be culturally sensitive while mitigating stigma associated with being solely targeted.

A limitation of this study is a small sample that includes a specific segment of the African American community that is, at least in part, privileged compared to other segments of the same community, with regard to educational attainment. Additionally, the health belief model may not be the most comprehensive framework to guide analyses since the social construction of health (and health inequalities) is not just a matter of "belief". Other variables must be taken into account, and especially constraints (be they economic, social or cultural). This is especially true in the U.S. where healthcare costs are potentially very high, compared to other countries. While these factors could decrease the generalizability of findings, this work is still an important first step toward addressing existing disparities. Given that African Americans have a disproportionately higher risk of developing SLE and frequently display more rapid progression of disease with increased morbidity and mortality [4, 5, 10, 12, 15, 16, 39], increased lupus awareness is critical in this group. This could lead to earlier diagnosis and treatment, which has been associated with significantly better outcomes in patients with newly diagnosed lupus nephritis (LN) [40, 41] and minimized risk of inflammation-induced irreversible kidney damage [42].

Current epidemiologic data clearly document the existence of racial disparities in SLE. It also is evident that the scientific community does not completely understand why these disparities exist. Previous research has demonstrated significant gaps in knowledge about the signs, symptoms, and psychosocial effects of lupus [43]. In one study, public opinion leaders (POLs) provided anecdotes of encounters with individuals who thought that discoid lupus was contagious or that lupus was related to cancer or AIDS. Additionally, several POLs reported that the clinical staff (e.g., receptionist, triage nurses) they encountered during visits to their doctors often lacked basic knowledge about lupus and its clinical manifestations. POLs also shared that male lupus patients were often poorly represented or left out from lupus-centered events and initiatives due to the perception that lupus is “a woman’s disease.” Similarly, POLs reported that young people often declined invitations to attend support group meetings or to discuss their disease. Several POLs described observing a sense of invincibility that accompanies youth, which makes it difficult for young people to embrace the realities of their illness. Since the disease can be disfiguring, POLs have reported that young patients avoid interacting with older patients out of fear of seeing the way the disease manifests over time [33]. Working with the target community to identify and implement tools to enhance health education could advance understanding and address health disparities in lupus.

## Conclusion

Participants in our study stressed the necessity of lupus education and awareness among African Americans youth. Some participants encountered lupus only through family members who had been diagnosed with the disease. Still, they wanted resources that would enable them to advocate for themselves and their families. Given the early age of onset for lupus, it is therefore vital to include African American youth in increasing education and awareness about lupus. Traditional intervention models do not always include local youth advocates to elevate awareness and screening for lupus among their community. Therefore, the best advocates, or lupus liaisons, should be African American youth as opposed to experts to “meet individuals where they are” in their perceived understanding of lupus. To that end, future interventions should use evidence-based frameworks to develop best practices for implementing lupus awareness among AA youth. While findings suggest preference for youth advocates, partnerships should occur among AA youth community members (including students on HBCU campuses) and expert lupus advocates to elevate the significance of the benefits of preventive health practices.

## Supplementary Information

**Additional file 1: Fig. S1.** Health belief model. Rosenstock, I. M. (1974). Historical origins of the health belief model. Health education monographs, 2(4), 328–335.

## Data Availability

The datasets used and/or analyzed during the current study are available from the corresponding author on reasonable request.

## Abbreviations

HBCU: Historically Black College and University
SLE: Systemic lupus erythematosus
AA: African American
IMACT: Improving Minority Participation in Clinical Trials
HBM: Health Belief Model
IRB: Institutional Review Board
MUSC: Medical University of South Carolina
LN: Lupus nephritis
POL: Public opinion leader
