# Supplementary figures and images for: Formative research to promote lupus awareness and early screening at Historically Black College and University (HBCU) communities in South Carolina
Source: BMC Rheumatol. 2022 Dec 31;6:92. doi: 10.1186/s41927-022-00323-6 (PMC9805239; doi:10.1186/s41927-022-00323-6)

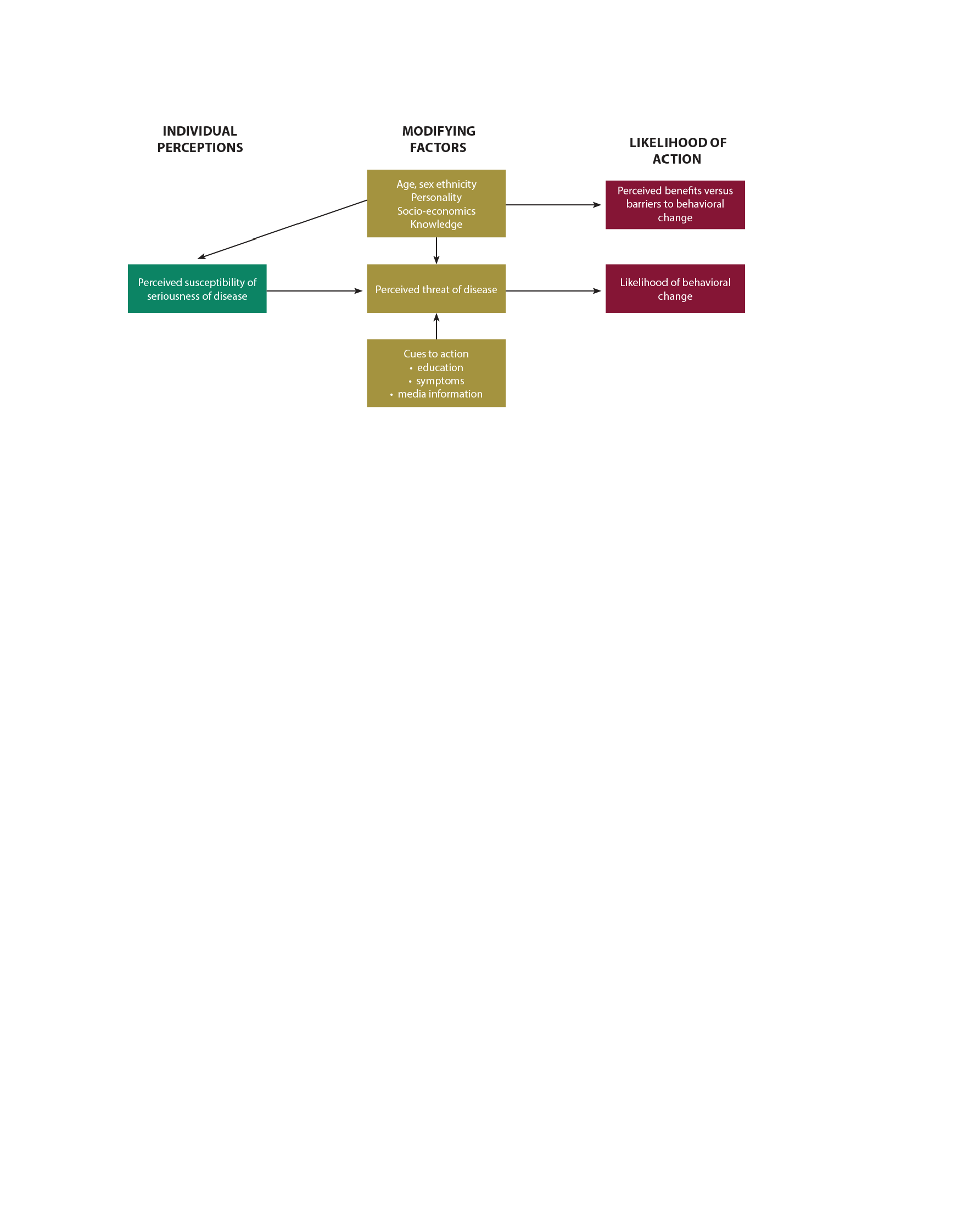

Supplement: Supplementary file 1 — Additional file 1: Fig. S1. Health belief model. Rosenstock, I. M. (1974). Historical origins of the health belief model. Health education monographs, 2(4), 328–335. [file 41927_2022_323_MOESM1_ESM.tiff]
